# Exploring the Regulation and Function of *Rpl3l* in the Development of Early-Onset Dilated Cardiomyopathy and Congestive Heart Failure Using Systems Genetics Approach

**DOI:** 10.3390/genes15010053

**Published:** 2023-12-29

**Authors:** Akhilesh K. Bajpai, Qingqing Gu, Buyan-Ochir Orgil, Neely R. Alberson, Jeffrey A. Towbin, Hugo R. Martinez, Lu Lu, Enkhsaikhan Purevjav

**Affiliations:** 1 Department of Genetics, Genomics and Informatics, University of Tennessee Health Science Center, Memphis, TN 38103, USA; abajpai3@uthsc.edu (A.K.B.); qgu4@uthsc.edu (Q.G.); 2 The Heart Institute, Le Bonheur Children’s Hospital, University of Tennessee Health and Science Center, Memphis, TN 38103, USA; borgil@uthsc.edu (B.-O.O.); nalberson@uthsc.edu (N.R.A.); jtowbin1@uthsc.edu (J.A.T.); hmarti31@uthsc.edu (H.R.M.); 3 Children’s Foundation Research Institute, Le Bonheur Children’s Hospital, Memphis, TN 38103, USA; 4 Cardiology, St. Jude Children’s Research Hospital, Memphis, TN 38105, USA

**Keywords:** ribosomal protein L3-like, dilated cardiomyopathy, BXD, systems genetics, immune infiltration

## Abstract

Background: Cardiomyopathies, diseases affecting the myocardium, are common causes of congestive heart failure (CHF) and sudden cardiac death. Recently, biallelic variants in ribosomal protein L3-like (RPL3L) have been reported to be associated with severe neonatal dilated cardiomyopathy (DCM) and CHF. This study employs a systems genetics approach to gain understanding of the regulatory mechanisms underlying the role of *RPL3L* in DCM. Methods: Genetic correlation, expression quantitative trait loci (eQTL) mapping, differential expression analysis and comparative functional analysis were performed using cardiac gene expression data from the patients and murine genetic reference populations (GRPs) of BXD mice (recombinant inbred strains from a cross of C57BL/6J and DBA/2J mice). Additionally, immune infiltration analysis was performed to understand the relationship between DCM, immune cells and *RPL3L* expression. Results: Systems genetics analysis identified high expression of *Rpl3l* mRNA, which ranged from 11.31 to 12.16 across murine GRPs of BXD mice, with an ~1.8-fold difference. Pathways such as “diabetic cardiomyopathy”, “focal adhesion”, “oxidative phosphorylation” and “DCM” were significantly associated with *Rpl3l*. eQTL mapping suggested *Myl4* (Chr 11) and *Sdha* (Chr 13) as the upstream regulators of *Rpl3l*. The mRNA expression of *Rpl3l*, *Myl4* and *Sdha* was significantly correlated with multiple echocardiography traits in BXD mice. Immune infiltration analysis revealed a significant association of *RPL3L* and *SDHA* with seven immune cells (CD4, CD8-naive T cell, CD8 T cell, macrophages, cytotoxic T cell, gamma delta T cell and exhausted T cell) that were also differentially infiltrated between heart samples obtained from DCM patients and normal individuals. Conclusions: *RPL3L* is highly expressed in the heart tissue of humans and mice. Expression of *Rpl3l* and its upstream regulators, *Myl4* and *Sdha*, correlate with multiple cardiac function traits in murine GRPs of BXD mice, while *RPL3L* and *SDHA* correlate with immune cell infiltration in DCM patient hearts, suggesting important roles for *RPL3L* in DCM and CHF pathogenesis via immune inflammation, necessitating experimental validations of *Myl4* and *Sdha* in *Rpl3l* regulation.

## 1. Introduction

The most common form of cardiac muscle disease in pediatric patients is dilated cardiomyopathy (DCM), followed by hypertrophic (HCM), restrictive (RCM), LV noncompaction (LVNC) and mixed cardiomyopathies [1]. Recently, several novel biallelic variants in the *RPL3L* (ribosomal protein L3-like) gene have been reported in neonates and infants with early-onset severe DCM and congestive heart failure (CHF), typically requiring heart transplantation [2,3,4,5]. Of note, the severity of left ventricular (LV) dilation and ventricular dysfunction at listing for heart transplant is significantly associated with outcome in infants and young children with early-onset DCM [6]. Therefore, understanding the pathogenesis and risks of severe LV dilation and early manifestations of DCM is crucial for therapeutic management of neonatal and infant DCM cases. 

The *RPL3L* gene encodes the 60S ribosomal protein that is located on chromosome (Chr) 16p13.3 near the *PKD1* (polycystic kidney disease 1) and *TSC2* (TSC complex subunit 2) genes [7]. The RPL3L protein is composed of 407 amino acids and has 77% identity to RPL3 (ribosomal protein L3), a highly conserved and ubiquitously expressed protein of the 60S subunit of ribosomes that mediates and controls protein translation in many organs [7,8]. In contrast to RPL3’s vital roles in most mammalian tissues [9], RPL3L is exclusively and highly expressed in skeletal and cardiac muscle tissues [10]. Alongside the development of early-onset severe DCM associated with biallelic mutations, missense and splice donor *RPL3L* variants such as p.Val262Met, p.Ala75Val and c.1167+1G>A have also been associated with prolonged electrocardiographic P-wave duration and atrial fibrillation (AF) [11,12], while the p.R242W variant has been identified as a modifier for catecholaminergic polymorphic ventricular tachycardia (CPVT) seen in siblings carrying a homozygous agrin (*AGRN*) gene variant [13]. In mice, RPL3L has been experimentally linked to skeletal muscle dystrophy in mdx mice, a model of Duchene muscular dystrophy (DMD) [14]; knockdown of RPL3L expression in the tibialis anterior of mdx mice resulted in increased muscle force, suggesting that a decrease in endogenous RPL3L may have a protective role in DMD [15]. Moreover, RPL3L has been predicted to negatively regulate skeletal muscle growth and size based on the finding that specialized ribosomes containing RPL3L altered the translational activity. 

Several groups have recently reported mouse models with ablated *Rpl3l* engineered on different genetic backgrounds, providing insights into its biological roles and functions [10,16,17]. While these knockout *Rpl3l*^−/−^ strains have shown variable cardiac phenotypes, all studies observed a compensatory increase in RPL3 in response to *Rpl3l* ablation. For example, *Rpl3l*^−/−^ mice, created on C57BL/6NTac (Taconic) and C57BL/6 (JAX) genetic backgrounds by Grimes et al. [16], showed no apparent cardiac dysfunction or remodeling at baseline or after pressure overload, while these knockouts exhibited a significant reduction in heart weight while aging. Another group also showed no significant pathological traits of heart or skeletal muscles in their C57BL/6J (JAX) *Rpl3l^−/−^* mice, but a significant increase in total lean mass was observed in their aged knockouts [10]. These results were explained by a compensatory increase in RPL3, which, in turn, modulates the localization of ribosomes and mitochondrial activity in cardiomyocytes. Shiraishi et al., however, reported a cardiac phenotype with impaired cardiac contractility and increased LV wall thickness in their *Rpl3l*^−/−^ mice created in the same C57BL/6J (JAX) strain [17]. Although the reason for the disparity of phenotypes seen in these knockout mice remains uncertain, the authors showed that RPL3L regulates ribosome occupancy at mRNA codons and that ablation of *Rpl3l* was linked to translation elongation dynamics for transcripts related specifically to cardiac muscle contraction and DCM [17]. 

Due to the inconsistency of phenotypes observed by these different groups, we conducted a comprehensive analysis employing a systems genetics approach to understand the regulatory mechanisms underlying severe DCM cases with CHF associated with *RPL3L*. We hypothesized that different genetic backgrounds affect the expression of *RPL3L*, and elucidation of gene–gene interactions using reference populations with diverse genetic backgrounds would be advantageous in understanding the regulatory mechanisms of *RPL3L*. In this study, we utilized the BXD family of mice derived from C57BL/6J and DBA/2J strains that has recently been demonstrated to be an ideal murine genetic reference population (GRP) for dissecting the genetic determinants and mechanisms involved in the pathogenesis of cardiomyopathies and the failing heart [18]. We employed a comparative mouse–human genetic analysis, and this analysis suggested that *Rpl3l* is upstream-regulated by *Myl4* and *Sdha*. Furthermore, the results of our study revealed that *Rpl3l* and *Sdha* are involved in the development of DCM and CHF via immune cell infiltration and inflammation. 

## 2. Materials and Methods

### 2.1. Mouse BXD Family and Heart Gene Expression Data

Systems genetics methods were employed on forty male BXD mice and their parental strains, C57BL/6J and DBA/2J, for generating the heart gene expression data. Details of tissue collection, RNA isolation and Affymetrix Mouse Gene 2.0 ST microarray analysis were described previously [18,19]. Murine heart gene expression data used in the current study were generated by our collaborative efforts at the University of Tennessee Health Science Center (UTHSC) and are publicly accessible through the GeneNetwork (http://genenetwork.org/, accessed on 8 August 2022) with accession #GN485 (EPFL/LISP BXD CD Heart Affy Mouse Gene 2.0 ST Gene Level (Jan14) RMA). All mice were fed a chow diet (6% kcal/fat, 20% protein, 74% carbohydrate) and euthanized at 29 weeks of age under isoflurane anesthesia following an overnight fast. The heart tissues were taken immediately, weighed and then frozen in liquid nitrogen. All animal procedures were approved by IACUC of UTHSC and by the Swiss cantonal veterinary authorities of Vaud under licenses 2257.0 and 2257.1.

### 2.2. Array Profiling and Data Analysis

Briefly, raw microarray data files were first RMA (robust multichip array) normalized [20] and then log2 transformed and Z normalized as previously described [21]. Further, instead of leaving the mean at zero and the standard deviation of one unit, we up shifted to a mean of 8 units and increased the spread by having a standard deviation of 2 units (2Z + 8 normalization). This was undertaken to remove negative values. 

### 2.3. Correlation Analysis

The correlation between *Rpl3l* expression and that of other genes in BXD heart dataset, GN485 (EPFL/LISP BXD CD Heart Affy Mouse Gene 2.0 ST Gene Level (Jan14) RMA) and gene–heart phenotype correlation was calculated using the Pearson correlation method. The correlation values with a *p*-value < 0.05 were considered significant. Furthermore, among the correlated genes, those having a mean expression > 7 were considered for further analysis. The RNA-seq expression data from the GTEx database [22] were used to derive the *RPL3L*-correlated genes in the human heart with a correlation *R* threshold of 0.5 and *p*-value threshold of 0.05.

### 2.4. Expression Quantitative Trait Locus (eQTL) Mapping

We used the interval mapping method to perform eQTL mapping through the webQTL tool on GeneNetwork (http://gn1.genenetwork.org/webqtl/main.py, accessed on 5 November 2023) to identify the genomic loci associated with regulation of *Rpl3l* expression. Interval mapping employed the likelihood ratio statistics (LRS) to measure the linkages between the investigated trait and genotype markers. This method used approximately 7300 informative SNP genotype markers across the BXD mouse genome for calculating the association with *Rpl3l* mRNA expression variation. Furthermore, the genome-wide significant (LRS = 17.40) and suggestive (LRS = 10.85) thresholds were determined using 1000 permutation tests.

### 2.5. Candidate Gene Selection

We used a 1.5-LOD confidence interval for shortlisting the potential candidate genes associated with *Rpl3l* expression regulation in BXD mice hearts. For this, a scoring system ranging from 0 to 10 was employed to prioritize the genes in the selected QTL regions. Our scoring system included five different parameters, each of which was assigned a different weight based on its overall significance. These parameters were mainly from two sources: our GeneNetwork portal and publicly available functional/pathway-related databases. The parameters and their respective weights have been scored according to the following criteria: (1) expression in the heart and assigning a score of 1 to the genes located in the QTL interval with a mean expression of ≥8 in the heart tissue; (2) coding sequence DNA variants and assigning a score of 1 to the genes harboring one or more variants, such as non-synonymous, frame shift and stop gain or loss (we used our previously generated whole-genome sequencing (WGS) data of the two parental strains, C57BL/6J and DBA/2J) [23]; (3) *cis*-regulation and assigning a score of 1 to the genes located within 10 Mb flanking regions of the peak SNP (for this, we performed eQTL mapping for the all genes located within the QTL interval to evaluate their regulation); (4) gene-trait correlation using the Pearson correlation method on GeneNetwork (www.genenetwork.org, accessed on 20 December 2022) [24] and assigning a score of 2 to the genes located within the QTL interval with a significant (*p* < 0.05) relationship with *Rpl3l* expression; and (5) functional relevance and assigning a score of 5. To determine the functional significance of the genes within the identified QTLs, we used publicly available databases pertaining to rodents including Mouse Genome Informatics (MGI, http://www.informatics.jax.org/, accessed on 18 August 2022) [25], International Mouse Phenotyping Consortium (IMPC, http://www.mousephenotype.org/, accessed on 18 August 2022) [26] and Rat Genome Database (RGD, www.rgd.mcw.edu, accessed on 18 August 2022) [27]. Furthermore, the following human databases were searched to obtain the associated genes: GWAS Catalog (www.ebi.ac.uk/gwas, accessed on 19 August 2022) [28], Kyoto Encyclopedia of Genes and Genomes (KEGG, https://www.genome.jp/kegg/, accessed on 19 August 2022) [29] and Alliance of Genome Resources (https://www.alliancegenome.org/, accessed on 19 August 2022) [30]. All of these databases were queried with the keyword “cardiomyopathy” and associated genes were downloaded. However, the “cardiomyopathy” term retrieved no phenotype in the IMPC database; therefore, it was queried with “cardiac”, and genes associated with all the resultant phenotypes were downloaded. Genes obtained from these resources were then matched with the QTL interval genes and assigned a specific score if present. Genes from RGD and the human GWAS Catalog were given a higher weight with a score of 2 for being directly involved in the causation of the disease. Meanwhile, genes from other functional resources were given a score of 1. The scores across all the five parameters were summed for each gene, and those with a total score of ≥5 (50% of the total score) were selected as potential candidates for further analysis.

### 2.6. RPL3L Expression in Human and Mouse

RNA sequencing-based expression of *Rpl3l* across various human and mouse tissues were obtained from publicly available gene expression databases. The human RNA-Seq data corresponding to 28 adult tissues were obtained from the GTEx database [22], a comprehensive public resource used to study tissue-specific gene expression and regulation (https://gtexportal.org/home/, accessed on 14 December 2023), whereas the mouse RNA-Seq data were obtained from the Mammalian Transcriptomic Database [31]. Heart cell type specific data corresponding to *RPL3L* expression were obtained from GTEx and the Human Protein Atlas (HPA) databases (https://www.proteinatlas.org/, accessed on 27 October 2023). The cell type-specific data in GTEx were generated through snRNA-seq across 8 human tissues [32], while in HPA, it was collected from multiple studies. Furthermore, *Rpl3l* expression levels in the hearts of BXDs and their founder strains, C57BL/6J and DBA/2J, were obtained from our GN485 dataset (EPFL/LISP BXD CD Heart Affy Mouse Gene 2.0 ST Gene Level (Jan14) RMA) deposited in the GeneNetwork portal.

### 2.7. Differential Expression between Human Cardiomyopathy and Control Samples

We used the publicly available gene expression microarray study GSE120895 [33] to identify genes differentially expressed between human cardiomyopathy and control samples. The study was composed of human endomyocardial biopsies from 47 patients with DCM and 8 individuals with normal LV size and ejection fraction (LVEF) as controls. The raw data *CEL* files profiled on an Affymetrix Human Genome U133 Plus 2.0 Array were downloaded from the NCBI-GEO database (https://www.ncbi.nlm.nih.gov/geo, accessed on 30 October 2023), and then background corrected and normalized using RMA in R [34]. The samples were then clustered based on the normalized probe-set intensities using hierarchical clustering (Euclidean distance method), and those not falling in the same sample group were excluded. Finally, a total of 35 human cardiac samples (30 DCM and 5 normal) were considered for differential expression analysis. Differentially expressed genes with a false discovery rate (FDR) corrected (Benjamini and Hochberg correction) *p*-value < 0.05 were considered as statistically significant. 

### 2.8. Functional Enrichment Analysis

The enrichment analysis of *Rpl3l*-correlated genes and differentially expressed genes between human cardiomyopathy and control samples was performed using the *clusterProfiler* R package [35] with default parameters to identify enriched KEGG pathways and gene ontology (GO) annotations. In addition, we performed mammalian phenotype ontology (MPO) enrichment analysis of *Rpl3l*-correlated genes using the WEB-based Gene SeT AnaLysis Toolkit (WebGestalt, http://www.webgestalt.org, accessed on 5 November 2023) [36]. This analysis uses a hypergeometric statistical test to result adjusted *p*-values and enrichment ratios. The *p*-values were adjusted to account for multiple comparisons using the Benjamini and Hochberg correction, and annotations with a *p*-value < 0.05 were considered statistically significant.

### 2.9. Immune Cell Infiltration Analysis

We performed immune cell infiltration analysis using ImmuCellAI (http://bioinfo.life.hust.edu.cn/ImmuCellAI, accessed on 13 November 2023) [37]. The RMA normalized expression values corresponding to DCM and normal human heart samples were uploaded onto ImmuCellAI, and abundances of 24 different immune cell types were estimated in each sample/group using the single sample gene set enrichment analysis (ssGSEA) algorithm. The significance of differential infiltration of the cell types between DCM and normal groups was calculated and values with *p* < 0.05 were considered significant. Furthermore, the expression of *Rpl3l* and its potential modulators was correlated with the infiltration score of immune cell types that were significantly different between DCM and normal groups using the Pearson correlation method. The correlation analysis was performed using the corrplot R package (https://github.com/taiyun/corrplot, accessed on 1 November 2023) and the correlation *R*-values with *p* < 0.05 were considered significant.

## 3. Results

### 3.1. Expression of RPL3L in Human and Mouse Heart

**Figure 1:**
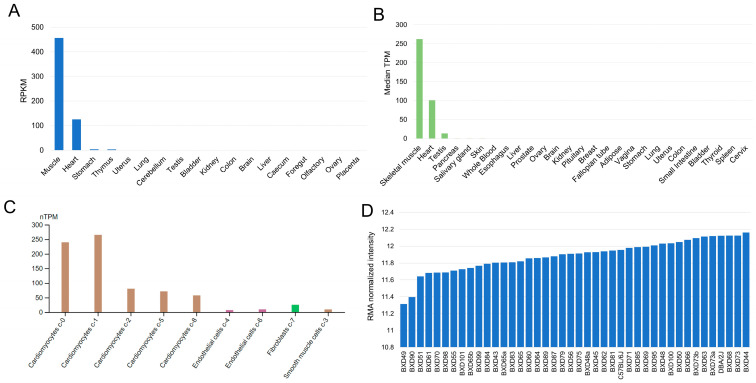
Expression of *RPL3L* in human and mouse. Expression levels of *RPL3L* (*y*-axis) across (**A**) mouse tissues, (**B**) human tissues, (**C**) different human heart cell type clusters and (**D**) the hearts of BXD and two parental strains (*x*-axis). The multi-tissue data (**A**,**B**) are based on bulk RNA sequencing results obtained from the Mammalian Transcriptomic Database (MTD: https://ngdc.cncb.ac.cn/mtd/, accessed on 14 December 2023) or GTEx database (https://www.gtexportal.org/, accessed on 14 December 2023), while the cell-type-specific expression data (**C**) are based on single-cell RNA sequencing and were obtained from the Human Protein Atlas database (https://www.proteinatlas.org/, accessed on 27 October 2023). The microarray expression data in BXD strains (**D**) were retrieved from GeneNetwork (https://genenetwork.org/, accessed on 8 August 2022) with the accession number: GN485. RPKM, reads per kilobase per million mapped reads; TPM, transcript per million; nTPM, normalized TPM.

Coupling mouse and human genome-phenome-wide association studies has been shown to be an efficient method by which to validate and translate key genotype–phenotype relations in human clinical cohorts [23]. We used publicly available data to investigate the expression levels of *RPL3L* at the mRNA level across multiple tissues of both human and mouse and across different cell types, including human heart cell types. While tissue expression was based on bulk RNA sequencing data, the cell-type-specific data were from single-cell RNA sequencing. Our results demonstrated that *RPL3L* is specifically expressed in the skeletal muscle and heart of both species (Figure 1). In mouse skeletal muscle and heart, the *Rpl3l* level was detected to be 456.9 and 125.3 reads per kilobase per million mapped reads (RPKM), respectively, whereas in other tissues, its mean expression was 0.6, with only two other tissues (stomach and thymus) showing an expression of >1 RPKM (Figure 1A). In the human heart, the median transcript per million (TPM) level of *RPL3L* was found to be 101 compared to its mean expression of 0.7 in other adult tissues, except in skeletal muscle based on bulk RNA sequencing data. Additionally, other than heart and skeletal muscle, *RPL3L* was detected only in testis with a median TPM of >1 (value = 13.82) and had <1 TPM in all other human tissues (Figure 1B). Furthermore, *RPL3L* was found to be enriched in myocytes with a higher fraction of cardiomyocytes exhibiting its mRNA expression (Figure 1C and Supplementary Figure S1). It should be noted that the gene is heart-enriched, not only based on mRNA levels but also based on its protein expression (Supplementary Figure S2). 

For clarifying the feasibility of using this systems genetics approach for elucidating the biological functions of *RPL3L*, we explored further the expression of *Rpl3l* in 40 strains of the BXD family and their parental strains, C57BL/6J (JAX) and DBA/2J, that were utilized as GRPs. Our results indicated a highly consistent expression of *Rpl3l* in the heart tissue of these BXD strains. The expression values ranged from 11.31 in BXD49 to 12.16 in BXD44, indicating an approximately 1.8-fold difference in *Rpl3l* expression across the BXDs. Further, between the parental strains, DBA/2J had comparatively higher (12.12) *Rpl3l* expression than the C57BL/6J strain (11.96) (Figure 1D). Thus, our analysis revealed that *Rpl3l* is specifically expressed in cardiomyocytes, while having very low or no expression in other cell types and tissues, suggesting its important physiological and pathological roles in mammalian heart. Moreover, *Rpl3l* exhibits expression variation in the heart tissue of BXD mice, hence these strains can be used for studying the detailed pathophysiological and functional roles of this gene [18]. 

### 3.2. Phenotypes and Pathways Associated with Rpl3l-Correlated Genes

It has been well-established that most of the genes perform their function through other partner genes either by directly interacting with them or through indirect associations. Hence, these sets of genes involved in similar functions are often co-regulated in a specific tissue or cell type. Thus, to understand the role of *Rpl3l* in the heart tissue and cardiomyopathy, we identified genes that are significantly correlated with *Rpl3l* expression in the heart of BXD mice using the GeneNetwork database. Our correlation analysis identified a total of 2895 genes to be significantly correlated with cardiac *Rpl3l* expression with a *p*-value < 0.05. Among these, 2241 genes had comparatively higher expression in the heart tissue of BXD mice with a mean expression level of >7. We used these genes for functional analysis to identify significantly enriched pathways and phenotypes, which may help us in gaining better insights into the mechanisms underlying *Rpl3l* function in BXD mice heart. 

**Figure 2:**
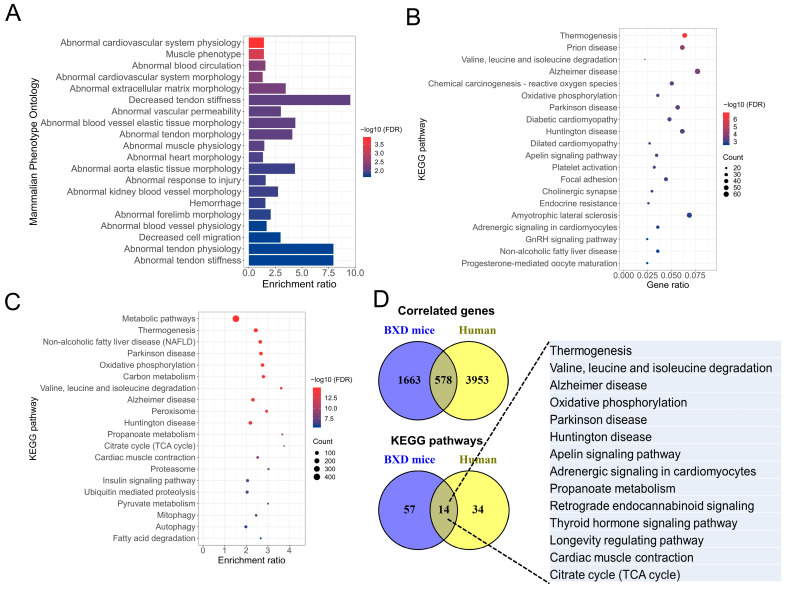
Functional enrichment analysis of *Rpl3l*-correlated genes. Top 20 (**A**) mammalian phenotype ontologies (MPOs) and (**B**) KEGG pathways significantly enriched (FDR *p*-value < 0.05) by *Rpl3l*-correlated genes in BXD mice. (**C**) KEGG pathways significantly enriched (FDR *p*-value < 0.05) by *RPL3L*-correlated genes in human. (**D**) *RPL3L*-correlated genes and KEGG pathways common between human and BXD mice. The *x*-axis indicates the gene/enrichment ratio, while the *y*-axis represents the MPOs/pathways. FDR, false discovery rate, is color-coded. KEGG, Kyoto Encyclopedia of Genes and Genomes.

The enrichment analysis showed that *Rpl3l*-correlated genes are primarily involved in MPOs related to abnormal cardiovascular physiology and morphology (Figure 2A). A total of 28 MPOs were significantly enriched with an FDR *p* < 0.05 by the correlated genes. “Abnormal cardiovascular system physiology” was the most significant MPO (FDR *p* = 1.24 × 10^−4^) with a total of 208 correlated genes and, among the significantly enriched MPOs, the highest number of genes (*n* = 240) were associated with “abnormal cardiovascular system morphology” (FDR *p* = 0.0048). The other significant MPOs included “abnormal heart morphology” (*n* = 178; FDR *p* = 0.0115), “abnormal blood circulation” (*n* = 100; FDR *p* = 0.0048), “muscle phenotype” (*n* = 186; FDR *p* = 5.75 × 10^−4^) and “abnormal heart ventricle morphology” (*n* = 92; FDR *p* = 0.0291). A complete list of significantly enriched MPOs is provided in Supplementary File S1.

Pathway analysis identified significant enrichment of 71 KEGG pathways by *Rpl3l*-correlated genes, and the top 20 of these are shown in Figure 2B. Thermogenesis was the most significant pathway (FDR *p* = 1.15 × 10^−7^) with 52 of the *Rpl3l*-correlated genes involved in it. Two important pathways, “diabetic cardiomyopathy” and “DCM” were in the list of top 20 significant pathways. While “diabetic cardiomyopathy” included 39 of the correlated genes (FDR *p* = 0.00045), 22 *Rpl3l*-correlated genes were involved in the “DCM” pathway (FDR *p* = 0.00078). The other interesting KEGG pathways that were significantly associated with the correlated genes were “platelet activation” (*n* = 26; FDR *p* = 0.00097), “adrenergic signaling in cardiomyocytes” (*n* = 29; FDR *p* = 0.0016), “MAPK signaling pathway” (*n* = 44; FDR *p* = 0.0056), “protein processing in endoplasmic reticulum” (*n* = 26; FDR *p* = 0.033) and “cardiac muscle contraction” (*n* = 16; FDR *p* = 0.0265). A list of all significantly enriched KEGG pathways is provided in Supplementary File S1.

To compare whether these results are consistent when human-correlated genes are used, we derived *RPL3L*-correlated genes for human heart tissue using GTEx RNA-seq expression data. The correlation analysis resulted in 4531 genes that were significantly and highly correlated with *RPL3L* mRNA expression in the human heart. Furthermore, pathway analysis of these genes resulted in 48 enriched KEGG pathways (FDR *p* < 0.05) with important pathways such as “cardiac muscle contraction”, “oxidative phosphorylation” and “citric acid cycle” in the top 20 (Figure 2C). When we compared the *RPL3L*-correlated human genes with those of BXD mice, 578 genes were found to overlap between both the species (Figure 2D). The overlap of the pathways between human and BXD mice was even more interesting. There were 14 pathways that were found to be commonly regulated by *Rpl3l*-correlated genes in both species (Figure 2D). Interestingly, several of these pathways are significant in the context of heart physiology, including “adrenergic signaling in cardiomyocytes” (*n* = 51; FDR *p* = 7.33 × 10^−4^), “cardiac muscle contraction” (*n* = 42; FDR *p* = 6.29 × 10−^9^) and “apelin signaling pathway” (*n* = 42; FDR *p* = 0.043). Thus, these results are suggestive of a similar mechanism of action by *RPL3L* in human and mice. Supplementary File S2 contains all significant pathways enriched by *RPL3L*-correlated genes in humans.

### 3.3. Differentially Expressed Genes and Functions between Human Cardiomyopathy and Control Samples

**Figure 3:**
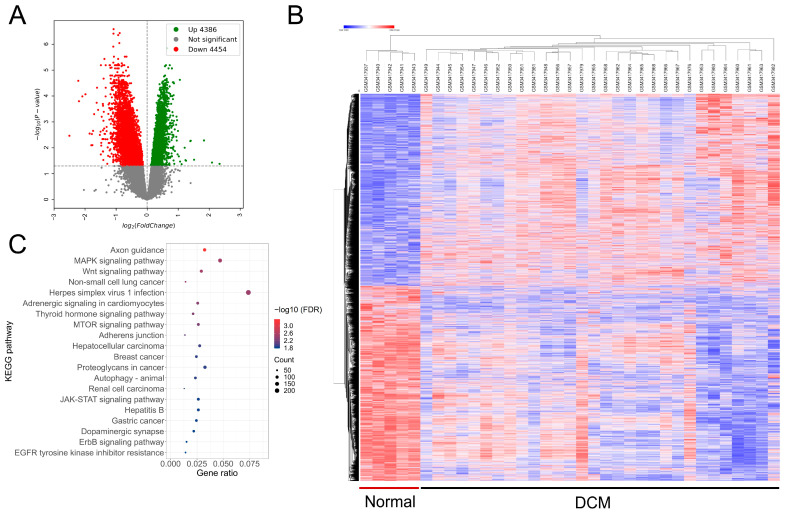
Differentially expressed genes between dilated cardiomyopathy (DCM) and normal human heart samples and enriched pathways. (**A**) Volcano plot representing the genes (*x*-axis) significantly differentially expressed (FDR-adjusted *p* < 0.05) between human DCM and normal samples (*y*-axis). (**B**) Heatmap representing the expression pattern of up- and down-regulated genes in each sample. Each column represents a sample, while each row represents a gene. Red color indicates high expression, whereas blue color indicates low expression of a gene in a specific sample. A complete list of significantly differential genes is provided as Supplementary File S3. (**C**) Top 20 KEGG pathways significantly represented (FDR adjusted *p* < 0.05) by the differentially expressed human genes. A complete list of all the significant pathways can be found in Supplementary File S4.

The analysis of *Rpl3l*-correlated genes in BXD mice and humans revealed the enrichment of multiple pathways that were associated with *Rpl3l* gene function in heart. Next, we wanted to narrow down the pathways that are involved in the pathogenesis of cardiomyopathy. A microarray gene expression GSE120895 study [33] was used to identify differentially expressed genes between human DCM and normal samples. The study contained a total of 55 samples, and clustering of these samples based on normalized gene expression data indicated 20 samples to be heterogenous in nature. Hence, we removed these 20 samples and used the remaining 35 specimens (30 DCM and 5 normal) to identify 8840 differentially expressed genes with an FDR-corrected *p*-value < 0.05 between the disease and control groups (Figure 3A). 

The expression pattern of these genes across DCM and normal samples is shown in Figure 3B. A complete list of the significantly differentially expressed genes is provided in Supplementary File S3. To understand the function of these genes, we performed pathway and gene ontology enrichment analysis. Our results showed the enrichment of various signaling pathways and a few cardiac-related pathways by the differential genes (Figure 3C and Supplementary File S4). The important pathways include the “MAPK signaling pathway” (*n* = 150; FDR *p* = 0.003), “adrenergic signaling in cardiomyocytes” (*n* = 82; FDR *p* = 0.005), “mTOR signaling pathway” (*n* = 84; FDR *p* = 0.006) and “autophagy-animal” (*n* = 75; FDR *p* = 0.013).

**Figure 4:**
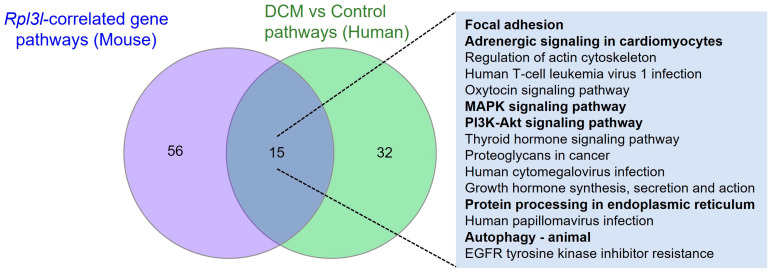
KEGG pathways common to *Rpl3l*-correlated genes in mice and differentially expressed genes in humans. The Venn diagram indicates the number of unique and common pathways between human and mouse gene-sets. The common pathways are listed in the adjacent box, and important pathways related to cardiac physiology/cardiomyopathy are highlighted in a bold font.

In addition, we compared the pathways that were enriched by the *Rpl3l*-correlated genes in mice and those significantly enriched by the differentially expressed genes in humans. The results indicated 15 KEGG pathways to be common between both human and mouse gene sets (Figure 4). 

Interestingly, key pathways known to be associated with cardiac morphology and function, such as “focal adhesion”, “MAPK signaling”, PI3K-Akt signaling”, “adrenergic signaling in cardiomyocytes”, “protein processing in endoplasmic reticulum” and “autophagy-animal”, were found to be commonly regulated by both human and mouse genes; further, we determined whether the same genes are involved in these pathways in both of these species. Supplementary Tables S1–S6 show the common genes that are involved in these pathways in both species along with their differential data in humans and correlation significance values in BXD mice. The overlapping of several genes from these pathways suggests that many of the functionally important differentially expressed genes in humans are also correlated with *Rpl3l* in the hearts of BXD mice. 

### 3.4. Chromosomal Loci in a Mouse Genome Regulating Rpl3l Expression

**Figure 5:**
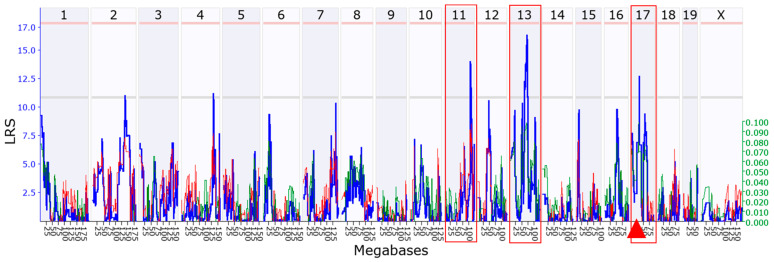
Manhattan plot showing chromosomal loci regulating *Rpl3l* expression. The *x*-axis denotes the chromosomal position in megabases on the mouse genome and the *y*-axis indicates the likelihood ratio statistic (LRS) score. The pink and grey horizontal lines indicate significant and suggestive LRS, respectively. The red triangle on the *x*-axis indicates the genomic position of *Rpl3l*. The LRS score is shown by the blue line, and additive effects are shown by red and green lines. Red boxes highlight the chromosomes harboring the suggestive eQTLs.

To identify genetic loci that are involved in *Rpl3l* expression regulation, we performed eQTL mapping using the interval mapping method on GeneNetwork. Our analysis identified three suggestive eQTLs. Two of them are located on Chr 11 at 104.768 Mb (peak LRS = 14) and on Chr 13 at 69.104 Mb (peak LRS = 16.4), respectively. These two loci are distantly located from *Rpl3l* (Chr 17 at 24.728 Mb), suggesting that they are *trans*-acting eQTLs. The other locus was close to the genomic location of the *Rpl3l* gene on Chr 17 (peak LRS = 12.7) and was defined as a *cis*-eQTL (Figure 5). Taken together, our mapping results suggest that *Rpl3l* is potentially regulated by sequence variants at multiple loci.

### 3.5. Identification of the Candidate Genes That Strongly Regulate Rpl3l Expression

The two trans-eQTLs with higher LRS scores detected via genetic mapping were considered for identifying the upstream regulators that may potentially regulate the expression of *Rpl3l*. We used a 1.5-LOD confidence interval to select the potential candidate genes. The 1.5-LOD interval on Chr 11 (104~111 Mb) and Chr 13 (58~76 Mb) contained 147 and 293 genes, respectively. Among these, 60 genes were significantly correlated with *Rpl3l* with 25 from Chr 11 and 35 from Chr 13. Of the 60 genes, 38 were highly expressed in BXD mouse hearts with a mean expression of ≥8. In addition, 21 genes contained coding sequence variants and 22 were *cis*-regulated. We then explored the functional relevance of these 60 genes using multiple resources pertaining to human, mouse and rat. The genes were then scored based on the correlation and genetic data obtained from the GeneNetwork database and functional data collected from the public resources. A total of 12 genes obtained a threshold score of 5 and were considered as candidates (Table 1). Chr 13 contributed most of the candidate genes (*n* = 9), while Chr 11 contributed 3 genes. Of the 12 genes, only three (*Myl4*, *Ace* and *Sdha*) were genetically (as well as functionally) associated with cardiomyopathy or cardiovascular physiology. *Myl4* (myosin, light polypeptide 4) and *Sdha* (succinate dehydrogenase complex, subunit A, flavoprotein) were the top two candidates, with scores of 7 and 6, respectively (Table 1). Both *Myl4* and *Sdha* are highly expressed in BXD mouse hearts with a mean expression of 10.8 and 14.6, respectively, are significantly correlated with *Rpl3l* and are functionally related to cardiomyopathy. Further, *Myl4* had nonsynonymous mutations in parental DBA/2J mice. Based on these results, we considered *Myl4* and *Sdha* to be strong candidate genes responsible for regulating *Rpl3l* expression in BXD hearts; hence, they may play a key role in *RPL3L* mutation-induced DCM.

### 3.6. Correlation of Rpl3l, Myl4 and Sdha with Cardiac Function Traits in BXD Mice

**Figure 6:**
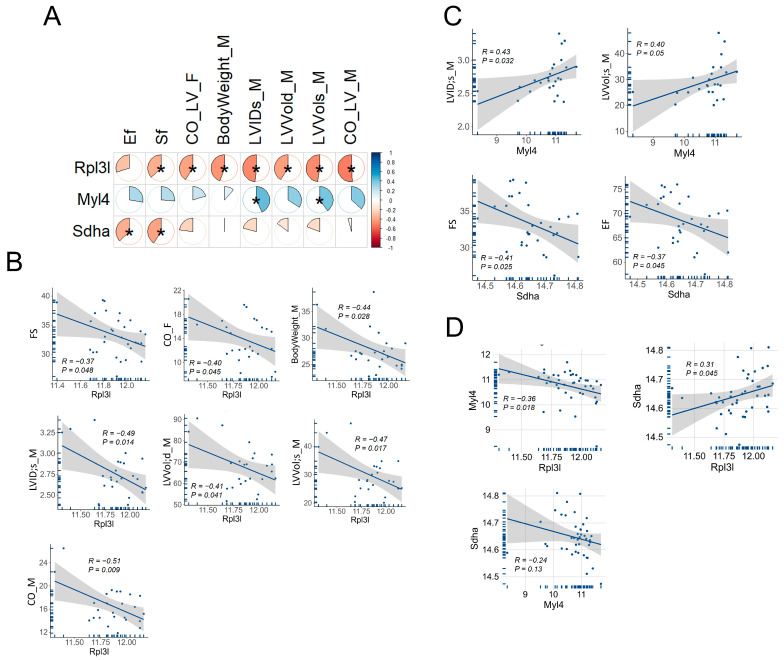
Correlation of *Rpl3l*, *Myl4* and *Sdha* expression with cardiac related phenotypes in BXD mice. The mRNA expression of the genes was correlated with cardiac trait values obtained from our GeneNetwork portal. Only phenotypes significantly correlated with at least one of these three genes are shown. (**A**) Correlation plot of genes with body weight and cardiac phenotypes. The significance of the correlation is indicated by an asterisk. The pie charts indicate the relative *R*-values. Correlations are coded by color and intensity, with red showing a positive correlation, and a negative correlation is shown in blue. (**B**) Scatter plots of individual correlations between *Rpl3l* and phenotypes. The *Rpl3l* gene expression is shown in the *x*-axis, whereas trait values are shown in the *y*-axis. The *R*- and *p*-values are shown within each plot. M, male; F, female; BodyWeight, body weight during echocardiography (g); EF, ejection fraction (%); FS, fractional shortening (%); CO, cardiac output (ml/min); LV, left ventricular; s, end-systole; d, end-diastole; ID, internal diameter (mm); Vol; volume (mg). (**C**) Correlation between *Myl4* and *Sdha* with cardiac phenotypes. The *R*- and *p*-values are shown within each plot. (**D**) Correlation between *Rpl3l*, *Myl4* and *Sdha*. The *R*- and *p*-values are shown within each plot.

We correlated the expressions of *Rpl3l*, *Myl4* and *Sdha* with cardiac phenotypes of BXD mice, retrieved from our GeneNetwork portal (Figure 6A). A total of 60 traits related to cardiovascular phenotypes were correlated with these genes. The results revealed that *Rpl3l* correlated with body weight and several important cardiac phenotypes, as identified via echocardiography of 17-week male (M) and female (F) BXD mice (Figure 6B). The *Rpl3l*-associated cardiac traits included measures of cardiac function, such as fractional shortening (FS), cardiac output (CO), LV end-systolic internal diameter (LVID;s) and LV volumes at end-systole and end-diastole (LVVol;s and LVVol;d, respectively) in BXDs. Interestingly, *Rpl3l* was negatively correlated with cardiac function (FS) and the volume of blood being pumped per minute by LV (CO) traits, suggesting a significant link between high *Rpl3l* expression and cardiac dysfunction. Moreover, a negative correlation between *Rpl3l* and LV chamber internal diameter and volumes suggested important roles of *Rpl3l* in the development of LV dilation. In addition, we assessed the association of two candidate regulatory genes, *Myl4* and *Sdha*, with cardiac phenotypes. While *Myl4* was positively correlated with LVID and LVVol at end-systole in BXD mice, *Sdha* was negatively correlated with EF and FS (Figure 6C). These results suggest that *Myl4* has an opposing effect on the LV dilation to that of *Rpl3l*, whereas *Sdha* and *Rpl3l* have synergistic effects on cardiac function, evidenced by mutual correlations with EF and FS among BXD mice. This trajectory was further supported by a significant positive association between *Rpl3l* and *Sdha* and a significant negative association between *Rpl3l* and *Myl4* (Figure 6D). Of note, *Myl4* and *Sdha* were negatively correlated with each other, although the relationship between them was statistically insignificant.

### 3.7. Analysis of Immune Cell Infiltration between DCM and Normal Heart and Correlations with Gene Expression

**Figure 7:**
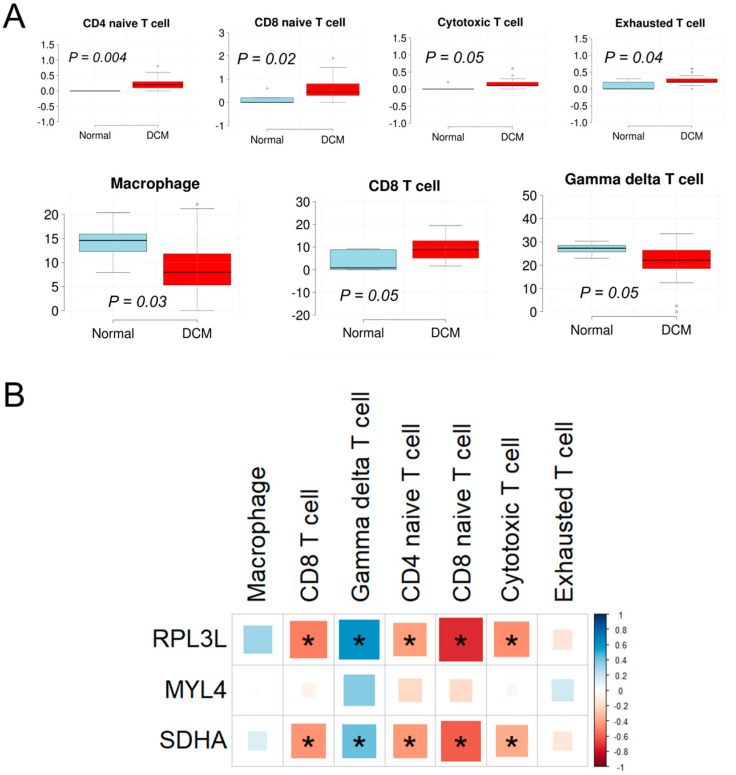
Immune cell infiltration in human heart samples and correlation with *RPL3L*, *MYL4* and *SDHA*. (**A**) Differential analysis of immune cell infiltration between dilated cardiomyopathy (DCM) and normal human heart samples. The *x*-axis indicates the sample groups, and the *y*-axis indicates the infiltration score. The analysis was performed for 24 different immune cell types using ImmuCellAI (http://bioinfo.life.hust.edu.cn/ImmuCellAI, accessed on 13 November 2023), and 7 cell types with significant differences are shown. The *p*-value is shown in each plot. (**B**) Correlation of *RPL3L* and its modulators (*MYL4* and *SDHA*) with immune cell infiltration. The normalized expression of the genes was correlated with the immune cell infiltration scores obtained from ImmuCellAI, and association is color- and intensity-coded. Red, negative correlation; blue, positive correlation. A significant correlation is marked with an asterisk.

Activation of inflammatory pathways occurs during myocardial injury induced by numerous damaging genetic variants in cardiac functional genes. We demonstrated previously that these damaging variants are enriched in patients with acute myocarditis, suggesting strong associations between cardiac functional genes and inflammation [38]. Therefore, we estimated the infiltration of immune cells in DCM and normal human heart samples and then explored whether there is a correlation between their infiltration rate and expression of *RPL3L*, *MYL4* and *SDHA* genes. Our analysis revealed that of the 24 immune cells evaluated, the infiltration of 7 cell types was significantly different (*p* < 0.05) between the DCM and normal groups. Among these, the infiltration of CD4-naive T cell, CD8-naive T cell, cytotoxic T cell, exhausted T cell and CD8 T cell was higher in the DCM group compared to the normal samples, whereas that of the macrophage and gamma delta T cell was lower in the DCM group (Figure 7A). Furthermore, the median infiltration rate of macrophage, gamma delta T cell and CD8 T cell was >1% in either DCM or normal sample groups, while it was <1% for the remaining four cell types. Moreover, to verify whether infiltration rates of these cell types are associated with gene expression, we correlated the normalized mRNA expression of *RPL3L*, *MYL4* and *SDHA* with the infiltration scores of the cell types. The results showed that the mRNA levels of both *RPL3L* and *SDHA* significantly correlated with the infiltration scores of five (CD4-naive T cell, CD8-naive T cell, cytotoxic T cell, gamma delta T cell and CD8 T cell) out of the seven immune cell types that were different between DCM and normal heart, while no correlation was observed for *MYL4*. Notably, both *RPL3L* and *SDHA* significantly negatively correlated with CD4-naive T cell, CD8-naive T cell, cytotoxic T cell, and CD8 T cell, whereas they were positively correlated with gamma delta T cell (Figure 7B and Table 2). 

## 4. Discussion

Several novel biallelic variants in the *RPL3L* gene have recently been identified to be associated with severe DCM and CHF in young pediatric patients [3,39]. These data have led to extensive studies of animal modeling by different groups [10,16,17]; however, the molecular and epistatic mechanisms underlying the role of *RPL3L* in heart physiology and related diseases necessitate further explorations. Therefore, we used a systems genetics approach and comparative bioinformatic analysis between human and mouse populations in this study to investigate the function of *Rpl3l* in the heart and identify potential genetic modulators that may affect its expression and their associations with cardiac function-related phenotypes. In addition, we applied immune cell infiltration analysis to reveal whether *Rpl3l* and its modulators are correlated with the infiltration of different immune cells in human DCM. 

To gain better insights into the role of *Rpl3l* in the heart, we initially evaluated the functions and pathways associated with *Rp3l*-correlated genes in the mouse. Enrichment analysis found 28 MPOs to be significantly associated with *Rpl3l*-correlated genes, nearly 50% of which were found to be directly related to heart physiology and morphology. In addition, 71 KEGG pathways were significantly associated with these *Rpl3l*-correlated genes, including pathways primarily related to cardiac morphology (e.g., focal adhesion), cardiomyocyte functions (e.g., adrenergic signaling in cardiomyocytes, protein processing in endoplasmic reticulum) and pathologies (e.g., DCM, MAPK signaling, autophagy), known to affect cardiac function via various complex molecular or signaling processes [40,41,42,43]. To further filter the dysregulated pathways that are important in the development of DCM, we used publicly available gene expression data in human DCM and normal heart samples and identified differentially expressed genes followed by analysis of their enriched pathways and processes, which revealed 15 common pathways that were significantly enriched by *Rpl3l*-correlated genes in mouse and differential genes in human heart (Figure 4). We further highlighted “focal adhesion”, “adrenergic signaling in cardiomyocytes”, “MAPK signaling”, “PI3K-Akt signaling”, “protein processing in endoplasmic reticulum” and “autophagy” as the important common pathways involved in the development of DCM and CHF. Focal adhesion molecules including FAK (focal adhesion kinase) have been extensively studied in the context of heart physiology, cardiac hypertrophy and CHF and have been systematically reviewed by multiple groups [44,45]. The adrenergic receptor (β-AR) signaling pathway is altered in both failing and aging hearts; therefore, it is utilized as an effective diagnostic and therapeutic target [46]. Activated MAPKs (mitogen-activated protein kinases) in response to various external stimuli regulate cardiac gene expression by translocating from the cytoplasm to the nucleus and phosphorylating various transcriptional factors in cardiomyocytes [40]. One of the key driving factors of CHF progression is cardiac fibrosis, and phosphoinositol-3 kinase (PI3K)/Akt signaling has been shown to be involved in the formation of pathological cardiac fibrosis [41]. Endoplasmic reticulum (ER) stress and autophagy pathways also received significant attention due to their involvement in the development and progression of cardiovascular diseases and CHF [43,47]. Thus, we highlight some important roles for *Rpl3l* in the development of cardiomyopathy, as the results of our analysis have demonstrated a strong association of *Rpl3l*-correlated genes with these important pathways that are involved in the cardiac pathophysiology of the human and mouse, while the involvement of the *Rpl3l*-correlated genes in the development of DCM requires further confirmation through knock-in and knock-out experiments. 

Systems genetics analysis using BXD mice identified three eQTLs for *Rpl3l* expression, two of which were distantly located *trans*-eQTLs (Chr 11 and Chr 13), while one was *cis*-eQTL located on Chr 17. Through a multi-criterion filtering strategy, we identified *Myl4* and *Sdha* as strong upstream regulators of *Rpl3l* out of 12 candidates found in these three chromosomal loci. Human *MYL4* is expressed in atria and ventricles during cardiac development; however, its expression disappears in normal ventricular myocardium by birth but remains in the atria during adulthood [48]. A study by Peng et al. [49] underscored a key role of *MYL4* for atrial contractile, electrical and structural integrity via positive regulation of ATPase activity, enabling actin monomer binding. Variants in this gene were shown to cause progressive atrial fibrosis, leading to atrial cardiomyopathy and familial AF in humans and rats, while *MYL4* loss-of-function was shown to block autophagy flux in atrial cardiomyocytes [50,51]. The other candidate modulator of *RPL3L*, *SDHA*, encodes a flavoprotein, a major catalytic subunit of the succinate dehydrogenase (SDH) enzyme, which is a key complex II protein of the mitochondrial respiratory chain that links two important pathways of ATP production in mitochondria, the Krebs cycle and oxidative phosphorylation. *SDHA* is highly expressed in the heart, as well as several other tissues, including the brain, alimentary canal and the genitourinary system. *SDHA* mutations cause mitochondrial disease, such as Leigh syndrome (LS) and multisystem (ocular, cardiomyopathy and neurological) disorders, and result in a significant tissue-specific reduction in SDH enzymatic activity, which has been linked to significant clinical phenotypes [52,53,54]. In this study, we found a positive correlation between *Myl4* expression and LV internal diameter and volume in BXD mice, as well as that higher expression of *Sdha* is associated with reduced cardiac function (EF and FS), supporting their roles in the heart. Interestingly, of the 12 potential candidate *Rpl3l*-modulators identified, 5 with a score =5 belonged to the *Zfp* family of genes that encode ZNF (zinc finger) proteins. Due to their capacity to interact with DNAs, RNAs, poly-ADP-ribose (PAR) and many other proteins, this group of proteins play essential roles in cancer, neurodegeneration and heart diseases via regulation of transcription, DNA repair, protein degradation and numerous other pathways [55,56,57]. Although the functional and genetic analyses strongly suggest *Myl4*, *Sdha* and several *Zpfs* as the upstream regulators of *Rpl3l*, further experimental validation is warranted to establish their exact roles in *Rpl3l*-induced heart pathophysiology and related diseases. 

While viral myocarditis is a common cause of pediatric DCM, damaging cardiac gene variants are also enriched in patients with acute myocarditis, suggesting strong associations between cardiac genes and inflammation [38,58]. Therefore, we explored the association of various immune cells with human DCM and *RPL3L* expression. Our analysis revealed significant differential infiltration of seven immune cell types (CD4-naive T cell, CD8-naive T cell, cytotoxic T cell, exhausted T cell, macrophage, gamma delta T cell and CD8 T cell) between DCM and normal sample groups. A similar pattern in the immune cell enrichment in DCM and AF was reported by Gan et al. [59]. For example, the decrease in macrophages and the increase in CD4 and CD8 T cell infiltration observed by us in DCM patients compared to the normal group were also noted in their study. In addition, varied patterns of immune cell infiltration were reported in other cardiomyopathy subtypes, such as a substantial decrease in macrophages, monocytes, DC, Th1, Treg and plasma cells in HCM, while CD8+ T cells and basophils were significantly enriched in these HCM hearts [60]. Pediatric patients with *RPL3L* mutations developed severe DCM in the absence of comorbidities such as atherosclerosis, hypertension, myocardial ischemia or diabetes mellitus [1], highlighting a direct connection of *RPL3L* with DCM and immune-mediated inflammation. As infiltration of immune cells can directly cause irreversible cardiac damage and contribute to CHF, the results of our study point to the importance of further investigations targeting the immune system in the management of genetic-origin DCM. 

## 5. Conclusions

*RPL3L* is highly expressed in the heart tissue of humans and mice. *Myl4* and *Sdha* were found to be strong candidates that regulate the expression of *Rpl3l* in murine heart. *Rpl3l*, *Myl4* and *Sdha* correlated significantly with the echocardiography traits in BXD mice. Immune infiltration analysis suggested an association between the infiltration rate of various immune cells and *RPL3L* and *SDHA* expression, opening avenues for further experimental validations of potential regulatory roles for *Rpl3l*, *Myl4* and *Sdha* in cardiac pathophysiology and therapeutic targeting in DCM.

## Figures and Tables

**Table 1:** Candidate genes involved in the regulation of *Rpl3l* expression.

Gene Name	Gene ID	Chr (Mb)	Parameters Considered for Scoring						
			Mean Expression	Coding Variant	*Cis*-Regulation	Sig. Corr.	RGD Causal	Function	Total Score
***Myl4***	**17896**	**Chr11:104.550663**	**10.8**	**Nonsyn**	**--**	**Y**	**Y**	**Y**	**7**
*Ace*	11421	Chr11:105.967945	11.7	--	Y	Y	--	Y	5
*Kpna2*	16647	Chr11:106.988629	10.3	Nonsyn	Y	Y	--	--	5
*Golm1*	105348	Chr13:59.640163	9.8	Nonsyn	Y	Y	--	--	5
*Zfp367*	238673	Chr13:64.133022	8.3	Nonsyn	Y	Y	--	--	5
*Zfp712*	78251	Chr13:67.038594	8.6	Nonsyn	Y	Y	--	--	5
*Zfp759*	268670	Chr13:67.128226	8.7	Nonsyn	Y	Y	--	--	5
*Zfp874a*	238692	Chr13: 67.426259	8.2	Nonsyn	Y	Y	--	--	5
*Zfp729a*	212281	Chr13:67.617001	10.5	Nonsyn	Y	Y	--	--	5
*Mtrr*	210009	Chr13:68.56078	9.4	Nonsyn	Y	Y	--	--	5
***Sdha***	**66945**	**Chr13:74.322254**	**14.6**	**--**	**--**	**Y**	**Y**	**Y**	**6**
*Rhobtb3*	73296	Chr13:75.869537	10.0	Nonsyn	Y	Y	--	--	5

**Comments:** Important candidate genes based on overall scoring are highlighted in bold. Y, “Yes”, indicating the availability of specific information for a specific gene; Nonsyn, nonsynonymous variant; RGD, Rat Genome Database; Chr, chromosome; Mb, megabases; Sig. Corr., significant correlation.

**Table 2:** Correlation between gene expression and immune cell infiltration in dilated cardiomyopathy.

Immune Cell Types	*RPL3L*		*MYL4*		*SDHA*	
	*R*	*p*	*R*	*p*	*R*	*p*
Macrophage	0.30	1.06 × 10^−1^	−0.01	9.55 × 10^−1^	0.13	4.90 × 10^−1^
CD8 T cell	−0.52	3.56 × 10^−3^	−0.07	7.20 × 10^−1^	−0.45	1.35 × 10^−2^
Gamma delta T cell	0.60	4.06 × 10^−4^	0.36	5.02 × 10^−2^	0.42	1.93 × 10^−2^
CD4-naive T cell	−0.42	2.26 × 10^−2^	−0.21	2.75 × 10^−1^	−0.44	1.63 × 10^−2^
CD8-naive T cell	−0.74	3.56 × 10^−6^	−0.19	3.13 × 10^−1^	−0.61	3.72 × 10^−4^
Cytotoxic T cell	−0.46	9.75 × 10^−3^	−0.04	8.19 × 10^−1^	−0.38	4.03 × 10^−2^
Exhausted T cell	−0.14	4.54 × 10^−1^	0.18	3.45 × 10^−1^	−0.13	4.87 × 10^−1^

## Data Availability

The expression data corresponding to BXD mice are available from our GeneNetwork portal (http://genenetwork.org/, accessed on 24 November 2023) with the accession #GN485. All other data presented are available either in the main manuscript or Supplementary Material.

## Supplementary Materials

The following supporting information can be downloaded at https://www.mdpi.com/article/10.3390/genes15010053/s1.
