# Supplementary material for: Exploring the Regulation and Function of Rpl3l in the Development of Early-Onset Dilated Cardiomyopathy and Congestive Heart Failure Using Systems Genetics Approach
Source: Genes (Basel). 2023 Dec 29;15(1):53. doi: 10.3390/genes15010053 (PMC10815855; doi:10.3390/genes15010053)
Supplement: Supplementary file 1 [file genes-15-00053-s001.zip › Supplementary_Tables_Figures.pdf]

Supplementary Information

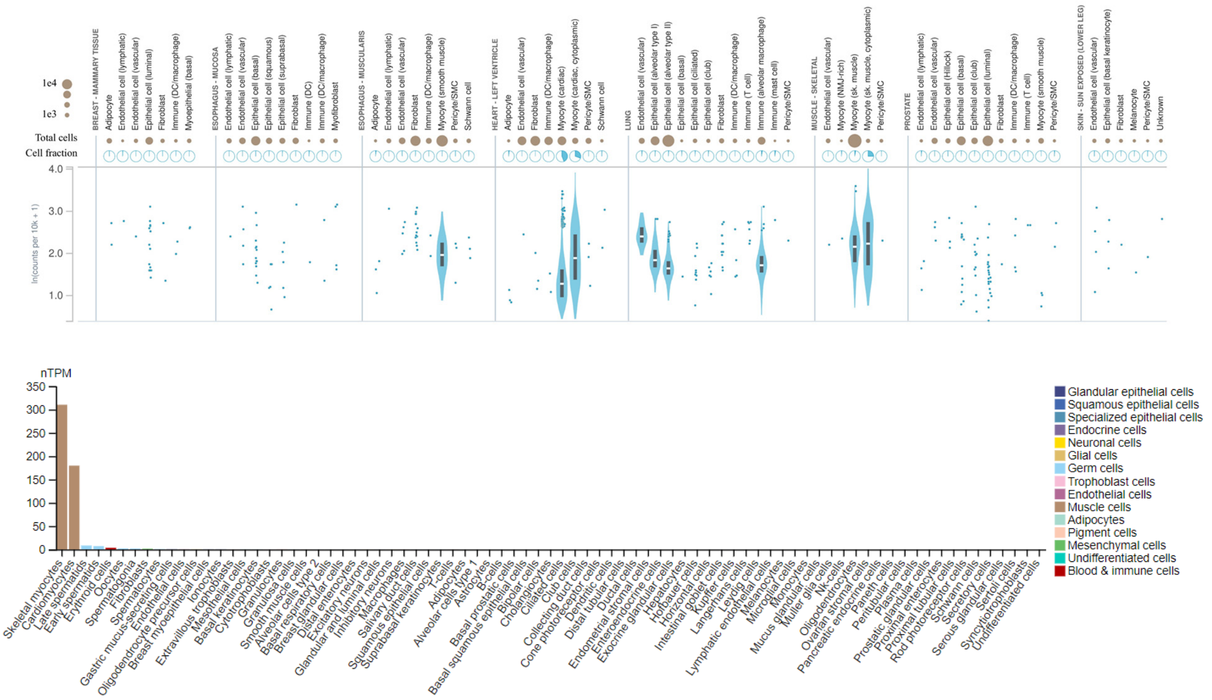

Supplementary Figure S1. *RPL3L* mRNA expression across human cell types. The cell-type specific expression data is based on single-cell snRNA sequencing and was obtained from (above) GTEx portal (https://gtexportal.org/home/gene/RPL3L) and (below) Human Protein Atlas database (https://www.proteinatlas.org/ENSG00000140986-RPL3L/single+cell+type), respectively. *RPL3L* is specifically expressed in myocytes. Furthermore, a higher fraction of heart myocytes shows the expression of the gene at mRNA level. Negligible expression of *RPL3L* is also exhibited by a few other cells.

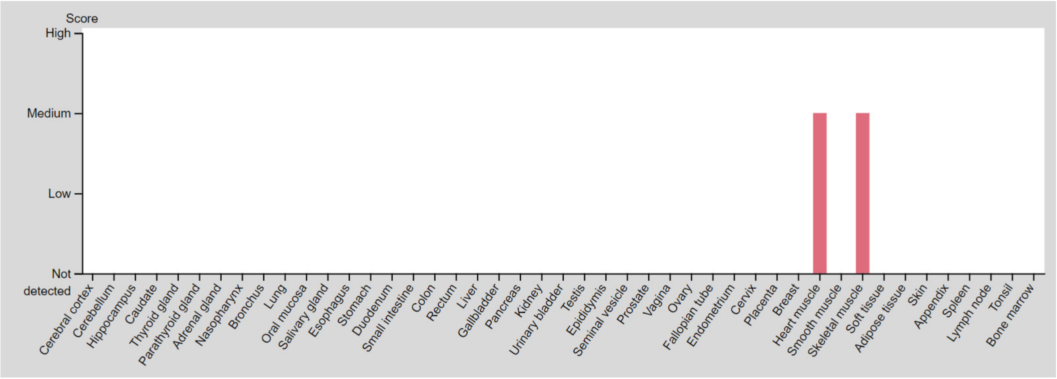

Supplementary Figure S2. RPL3L protein expression across human tissues. The protein level expression of *RPL3L* was obtained Human Protein Atlas database

(https://www.proteinatlas.org) and is based on immunohistochemical assays. As shown in the figure, RPL3L protein is specifically expressed in heart and skeletal muscle tissues.

Supplementary Table S1. 'MAPK signaling' pathway genes common between human and mouse

Symbol	Name	logFC [Human]	Abs fold [Human]	adj.P.Val [Human]	Sample p(r) [Mouse]
FLT1	fms related receptor tyrosine kinase 1	-0.64	-1.56	6.28E-05	0.0499
EGFR	epidermal growth factor receptor	0.49	1.40	0.00025	0.0103
PPM1A	protein phosphatase, Mg2+/Mn2+ dependent 1A	-0.56	-1.48	0.002391	0.0445
PPP3CC	protein phosphatase 3 catalytic subunit gamma	0.39	1.31	0.003197	0.0006
PPP5C	protein phosphatase 5 catalytic subunit	0.25	1.19	0.012586	0.0096
FGFR1	fibroblast growth factor receptor 1	-0.25	-1.19	0.012764	0.0002
ERBB3	erb-b2 receptor tyrosine kinase 3	0.33	1.25	0.030347	0.0269
CACNG6	calcium voltage-gated channel auxiliary subunit gamma 6	0.50	1.42	0.000241	0.0346
LAMTOR3	late endosomal/lysosomal adaptor, MAPK and MTOR activator 3	-0.61	-1.53	0.001723	0.0018
TNFRSF1A	TNF receptor superfamily member 1A	-0.53	-1.44	0.004441	0.0279
MKNK2	MAPK interacting serine/threonine kinase 2	-0.62	-1.53	0.004473	0.0076
TGFBR2	transforming growth factor beta receptor 2	-0.76	-1.70	0.00594	0.0006
IKBKB	inhibitor of nuclear factor kappa B kinase subunit beta	-0.46	-1.37	0.006658	0.0283
MAPKAPK5	MAPK activated protein kinase 5	-0.63	-1.54	0.007019	0.0096
PRKACA	protein kinase cAMP-activated catalytic subunit alpha	0.31	1.24	0.012752	0.0263
CACNA1H	calcium voltage-gated channel subunit alpha1 H	0.39	1.31	0.016581	0.0001
MAPK14	mitogen-activated protein kinase 14	-0.42	-1.34	0.024777	0.0459
AKT3	AKT serine/threonine kinase 3	-0.50	-1.41	0.026523	0.0062
CACNA2D1	calcium voltage-gated channel auxiliary subunit alpha2delta 1	0.24	1.18	0.031604	0.0463
FLNB	filamin B	-0.31	-1.24	0.033218	0.0098
GADD45G	growth arrest and DNA damage inducible gamma	-0.77	-1.71	0.047004	0.0271

Supplementary Table S2. 'Adrenergic signaling in cardiomyocytes' pathway genes common between human and mouse

Symbol	Name	logFC [Human]	Abs fold [Human]	adj.P.Val [Human]	Sample p(r) [Mouse]
CREB5	cAMP responsive element binding protein 5	-0.85	-1.80	0.002622	0.0199
ADCY1	adenylate cyclase 1	0.30	1.23	0.012066	0.0034

CACNG6	calcium voltage-gated channel auxiliary subunit gamma 6	0.50	1.42	0.000241	0.0346
PPP2CA	protein phosphatase 2 catalytic subunit alpha	-0.77	-1.71	0.000408	0.1612
CREB3L2	cAMP responsive element binding protein 3 like 2	0.33	1.26	0.000678	0.0040
ADCY7	adenylate cyclase 7	0.39	1.31	0.000871	0.0289
ADCY8	adenylate cyclase 8	0.43	1.35	0.002981	0.0497
ATP1B3	ATPase Na+/K+ transporting subunit beta 3	-0.48	-1.39	0.008923	0.0294
PRKACA	protein kinase cAMP-activated catalytic subunit alpha	0.31	1.24	0.012752	0.0263
ADCY2	adenylate cyclase 2	-0.26	-1.20	0.016076	0.0035
MAPK14	mitogen-activated protein kinase 14	-0.42	-1.34	0.024777	0.0459
AKT3	AKT serine/threonine kinase 3	-0.50	-1.41	0.026523	0.0062
TPM4	tropomyosin 4	-0.70	-1.63	0.031045	0.1457
CACNA2D1	calcium voltage-gated channel auxiliary subunit alpha2delta 1	0.24	1.18	0.031604	0.0463
ADRA1A	adrenoceptor alpha 1A	-0.52	-1.44	0.034298	0.0005
AGTR1	angiotensin II receptor type 1	-0.79	-1.72	0.009744	0.0274

Supplementary Table S3. 'Autophagy - animal' pathway genes common between human and mouse

Symbol	Name	logFC [Human]	Abs fold [Human]	adj.P.Val [Human]	Sample p(r) [Mouse]
RRAGC	Ras related GTP binding C	-0.61	-1.53	0.000643	0.0434
ATG10	autophagy related 10	0.33	1.26	0.003734	0.0001
PRKCQ	protein kinase C theta	0.36	1.28	0.00735	0.0250
PPP2CA	protein phosphatase 2 catalytic subunit alpha	-0.77	-1.71	0.000408	0.1612
WDR41	WD repeat domain 41	-0.56	-1.48	0.00298	0.0309
PRKACA	protein kinase cAMP-activated catalytic subunit alpha	0.31	1.24	0.012752	0.0263
AKT3	AKT serine/threonine kinase 3	-0.50	-1.41	0.026523	0.0062
CAMKK2	calcium/calmodulin dependent protein kinase kinase 2	0.25	1.19	0.04589	0.0338
HMGB1	high mobility group box 1	-0.47	-1.38	0.049242	0.0143

Supplementary Table S4. 'Protein processing in endoplasmic reticulum' pathway genes common between human and mouse

Symbol	Name	logFC [Human]	Abs fold [Human]	adj.P.Val [Human]	Sample p(r) [Mouse]
SSR3	signal sequence receptor subunit 3	-0.62	-1.54	0.00921	0.0284
CANX	calnexin	-0.59	-1.51	0.021533	0.0057
ATF6	activating transcription factor 6	-0.31	-1.24	0.023509	0.0347

DERL1	derlin 1	-0.61	-1.53	0.031392	0.2102
UBE2G1	ubiquitin conjugating enzyme E2 G1	-0.67	-1.59	0.0018	0.1310
SEC61G	SEC61 translocon subunit gamma	-0.82	-1.77	0.004346	0.0222
PDIA3	protein disulfide isomerase family A member 3	-0.50	-1.42	0.016309	0.0304
PRKN	parkin RBR E3 ubiquitin protein ligase	0.19	1.14	0.028373	0.0144
CKAP4	cytoskeleton associated protein 4	0.19	1.14	0.038451	0.0240
STT3B	STT3 oligosaccharyltransferase complex catalytic subunit B	-0.35	-1.28	0.040382	0.0172
STT3A	STT3 oligosaccharyltransferase complex catalytic subunit A	-0.44	-1.36	0.045837	0.0172

Supplementary Table S5. 'Focal adhesion' pathway genes common between human and mouse

Symbol	Name	logFC [Human]	Abs fold [Human]	adj.P.Val [Human]	Sample p(r) [Mouse]
FLT1	fms related receptor tyrosine kinase 1	-0.64	-1.56	0.00006	0.04985
EGFR	epidermal growth factor receptor	0.49	1.40	0.00025	0.01033
PARVA	parvin alpha	-0.54	-1.46	0.00129	0.02801
LAMA4	laminin subunit alpha 4	0.52	1.43	0.00010	0.01558
VWF	von Willebrand factor	0.41	1.33	0.00130	0.00011
COL1A1	collagen type I alpha 1 chain	0.35	1.27	0.00676	0.00110
ZYX	zyxin	0.54	1.45	0.01584	0.00144
LAMA5	laminin subunit alpha 5	-0.33	-1.26	0.01793	0.02276
ACTN1	actinin alpha 1	0.27	1.20	0.02479	0.00028
AKT3	AKT serine/threonine kinase 3	-0.50	-1.41	0.02652	0.00622
FLNB	filamin B	-0.31	-1.24	0.03322	0.00984
TLN1	talin 1	-0.41	-1.33	0.04639	0.01342

Supplementary Table S6. 'PI3K-Akt signaling' pathway genes common between human and mouse

Symbol	Name	logFC [Human]	Abs fold [Human]	adj.P.Val [Human]	Sample p(r) [Mouse]
CDK6	cyclin dependent kinase 6	-0.67	-1.59	0.00003	0.02976
FLT1	fms related receptor tyrosine kinase 1	-0.64	-1.56	0.00006	0.04985
EGFR	epidermal growth factor receptor	0.49	1.40	0.00025	0.01033
GNG4	G protein subunit gamma 4	0.33	1.25	0.00056	0.02995
CASP9	caspase 9	-0.46	-1.38	0.00073	0.04546
CREB5	cAMP responsive element binding protein 5	-0.85	-1.80	0.00262	0.01987
FGFR1	fibroblast growth factor receptor 1	-0.25	-1.19	0.01276	0.00022
MCL1	MCL1 apoptosis regulator, BCL2 family member	-0.49	-1.40	0.01944	0.04869

ERBB3	erb-b2 receptor tyrosine kinase 3	0.33	1.25	0.03035	0.02686
OSMR	oncostatin M receptor	-0.50	-1.42	0.04204	0.00755
LAMA4	laminin subunit alpha 4	0.52	1.43	0.00010	0.01558
PPP2CA	protein phosphatase 2 catalytic subunit alpha	-0.77	-1.71	0.00041	0.03263
YWHAZ	tyrosine 3-monooxygenase/tryptophan 5-monooxygenase activation protein zeta	-0.63	-1.54	0.00062	0.01604
CREB3L2	cAMP responsive element binding protein 3 like 2	0.33	1.26	0.00068	0.00396
VWF	von Willebrand factor	0.41	1.33	0.00130	0.00011
GNB1	G protein subunit beta 1	-0.48	-1.39	0.00426	0.03153
IKBKB	inhibitor of nuclear factor kappa B kinase subunit beta	-0.46	-1.37	0.00666	0.02835
COL1A1	collagen type I alpha 1 chain	0.35	1.27	0.00676	0.00110
LAMA5	laminin subunit alpha 5	-0.33	-1.26	0.01793	0.02276
AKT3	AKT serine/threonine kinase 3	-0.50	-1.41	0.02652	0.00622
